# Measuring inequalities of development at the sub-national level: From the human development index to the human life indicator

**DOI:** 10.1371/journal.pone.0232014

**Published:** 2020-04-30

**Authors:** Sergei Scherbov, Stuart Gietel-Basten

**Affiliations:** 1 World Population Program, International Institute for Applied Systems Analysis, Laxenburg, Austria; 2 International Laboratory of Demography and Human Capital, Russian Presidential Academy of National Economy and Public Administration, Moscow, Russian Federation; 3 Division of Social Science, The Hong Kong University of Science and Technology, Kowloon, Hong Kong; University of West London, UNITED KINGDOM

## Abstract

Despite being one of the most common measures of development, the Human Development Index [HDI] has been much criticized for its consistency, data requirements, difficulty of interpretation and trade-offs between indicators. The ‘Human Life Indicator’ [HLI] has been proposed as a ‘simple effective means’ of measuring development and, more specifically, as a viable alternative to the HDI. Reducing inequalities within countries is a core component of the Sustainable Development Goals; yet sub-national HDIs are subject to the same criticisms as national level indices (potentially more so). Our goal in this paper is to demonstrate ‘proof of concept’ in terms of the systematic application of the HLI to measure development at the subnational level. Using life tables for the United States of America, we calculate, for the first time, HLIs for each state for the period 1959–2016. This country was chosen for the comparatively long run of available sub-national life tables. We also calculate the extent to which mortality is distributed across the life course—a further measure of inequality and the role of the social determinants of health. The HLI clearly shows how striking regional inequalities exist across the United States. We find that HLI and HDI for the most recent time period are strongly correlated. The analysis demonstrates that HLI represents an effective means of measuring development at the sub-national level. Compared to HDI, HLIs are characterized by simpler calculation and interpretation; fewer data requirements; less measurement error; more consistency over time; and no trade-offs between components. A current challenge of producing sub-national HLIs is the lack of comprehensive civil registration and vital statistics systems in many parts of the Global South from which sub-national life tables can be generated. However, as more and more countries develop these systems the potential to produce HLIs will inevitably increase.

## Introduction

### The Human Development Index: Possibilities and challenges

Since 1990, the ‘Human Development Index’ [HDI] has been the core measure underpinning the UNDP’s *Human Development Reports* and has, more broadly, become a key figure for benchmarking development worldwide [1]. According to the UNDP, the HDI ‘was created to emphasize that people and their capabilities should be the ultimate criteria for assessing the development of a country, not economic growth alone’ [2]. As such, the HDI is ‘a summary measure of average achievement in key dimensions of human development: a long and healthy life, being knowledgeable and have a decent standard of living’ [2]. These three dimensions are calculated as a geometric mean of normalized indices of life expectancy at birth; mean of years schooling for adults aged 25 or more and expected years of schooling of children entering school age, and gross national income per capita [3].

As well as forming the heart of the influential *Human Development Reports*, the HDI is also widely applied elsewhere. It is often utilized in academic social, economic and demographic studies as a measure of general development which correlates to some other social indicator. For example, the influential 2009 paper by Myrskylä, Kohler, and Billari [4] posited an inverse relationship between fertility rates and ‘development’ based upon when a country crosses a given threshold of HDI. A 2017 study by Garcia-Tizon Larroca et al. [5], meanwhile, explored the extent to which HDI of maternal country of origin was a predictor of perinatal outcomes in Spain. A second major area is in terms of international benchmarking—especially in the popular press, and often in response to the publication of the *Human Development Reports* [6].

The HDI has, however, been the subject of much criticism and dispute. A first criticism is that given the empirically observed heterogeneity *within* countries, it is difficult to argue that a single number adequately reflects the different degrees of development across any given territory. Given that the ‘reduction of inequalities between and *within* countries’ is one of the key Sustainable Development Goals [SDGs] (emphasis added), understanding these differences at the sub-national level is of critical importance [7]. Some countries—especially larger ones—have seen the computation of sub-national HDIs precisely in order to gauge heterogeneity in development. In India, for example, the first sub-national HDIs were published in 1991, just one year after the first national figures were published [8]. Since then, other Indian organizations have calculated sub-national HDIs, including the Institute for Applied Manpower Research in 2007 [9] and by the UNDP itself in 2011 [10]. Similarly, a collaboration between UNDP China and the Institute for Urban and Environmental Studies at the Chinese Academy of Social Sciences produced principal-level HDIs for China for 2010 [11]. That study found that the national 2010 HDI of 0.693 (equivalent to Ecuador) did, in fact, mask a range of 0.569 (Tibet, akin to Morocco or Viet Nam) through to 0.821 (Beijing, akin to Slovakia or Malta) [12]. While such sub-national level studies do, indeed, shed great light on heterogeneity and inequalities *within* countries, there are not widespread and are difficult to compare given that different measurement procedures of HDI have been implemented over time—especially in 2010 and again in 2014 [13].

**Fig 1:**
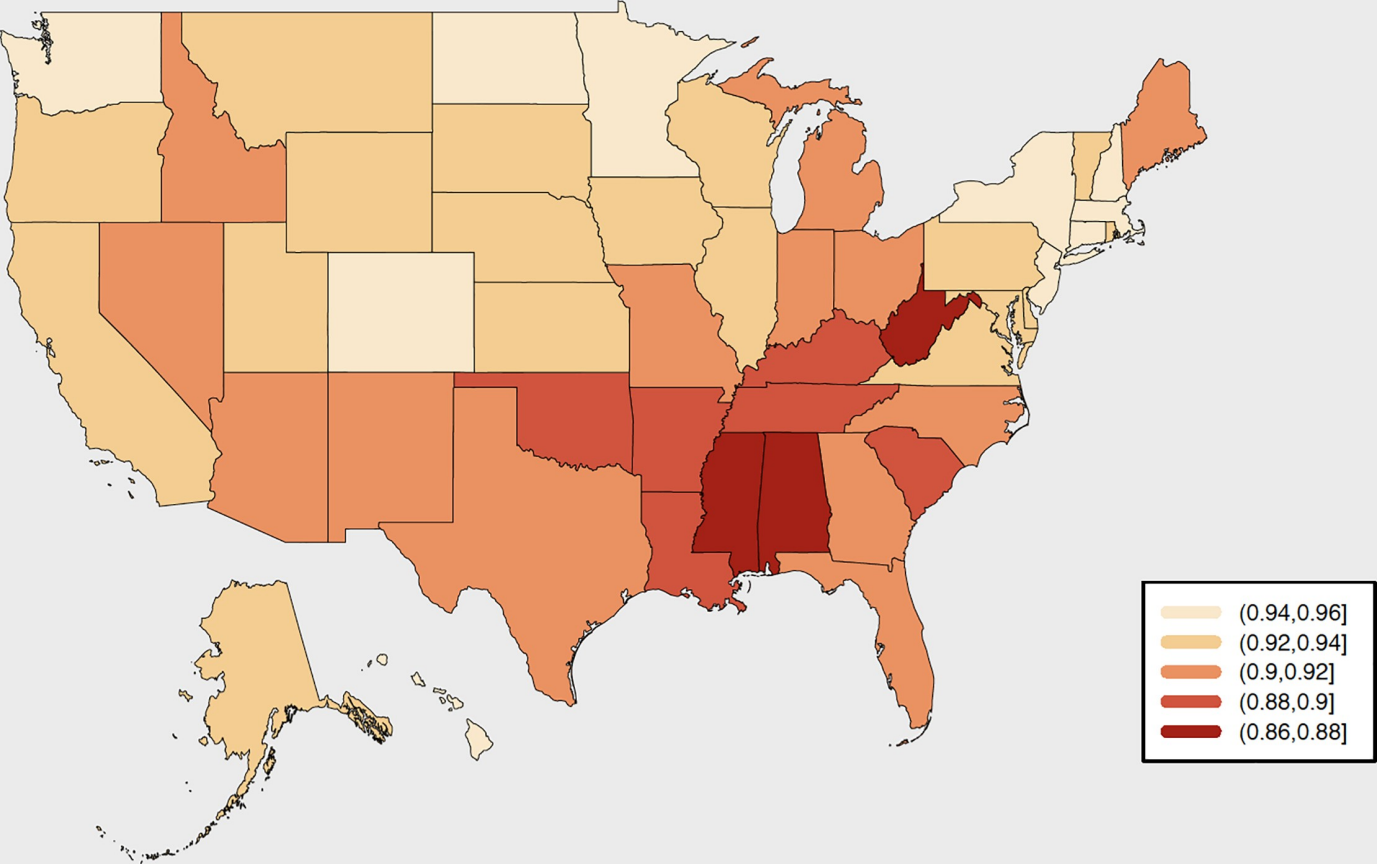
Sub-National HDIs for the states of the United States of America, 2016. Source: Map drawn by the authors based on data from [14].

To meet this criticism on a more systematic level, a team of researchers at Radboud University in the Netherlands have constructed a *Subnational Human Development Index* based upon the same indicators used to construct the national-level data [14,15]. Furthermore, these subnational indicators are constructed so that their national averages are equal to the national UNDP values. This dataset includes HDIs calculated for 161 countries and 1730 sub-national regions. Using this dataset we are able to get snapshots of regional inequalities in HDI from around the world. Fig 1, for example, is a representation of the sub-national differences in HDI for the states of the USA in 2016 [14]. Here, we see that the national figure of 0.923 again masks a wide heterogeneity between 0.866 (Mississippi, akin to Poland) through to 0.958 (Massachusetts, akin to Norway). These differentials clearly show that there are wide degrees of inequalities *within* countries at the regional level and do, indeed, give a clearer indicator of the task that lies ahead in terms reducing such inequalities as part of the SDG agenda—even in one of the most prosperous countries of the world.

The generation of these sub-national HDIs, therefore, adequately address a substantial criticism of the national figures—namely of the inability to adequately represent heterogeneity and inequality between regions. However, there are other criticisms of the measure which needs to be taken into account. These criticisms have been elucidated at length elsewhere [16–20] and a lengthy review is not required here. However, we might broadly group these critiques into four categories [21].

The first relates to the high degree of measurement error in each of the components [19]. This is especially the case in reference to the computation of GNI per capita [20]. Furthermore, because GNI per capita is only calculated periodically, there is a temporal mismatch between measures and any baseline errors or random variability is propagated through extrapolation until the next benchmark data are released [22].

A second issue relates to the consistency of the methods of calculation. As mentioned earlier, there were major changes in the method of calculating HDI introduced in 2010 and again in 2014 [13]. Clearly, this makes comparison over time difficult. While it is possible to generate a consistent time series of HDI by holding the functional form as well as the goalpost values, determining how these forms and values should be fixed is difficult, as is being sure of the actual historical values of GNI per capita using local currency prices. But more fundamentally, as Ghislandi et al. [21] argue, these changes ‘ultimately reflect the lack of consensus on the relative importance of the factors influencing human development’ (p.3)—namely should we be interested in the arithmetic or the geometric mean of school years? Or why should the cut-off for GNI per capita be above $75,000 rather than $65,000 or $85,000? Furthermore, in the same way, that we have seen various different changes to calculation mechanics to date, we may well assume that there will be further changes in the future causing further challenges to determining long-run time series and evaluating and benchmarking over time.

A third technical criticism concerns the implied trade-offs across the components of the HDI, such as the amount of GNI per capita required to make up for a decrease of one year in life expectancy. It has been observed that this has important policy implications. Based upon an analysis of Zimbabwe, for example, Ravallion [23] observed that the country would have returned a higher HDI in 2010 had it increased GNI per capita by just 0.52 PPP$ compared to a whole extra added year of life expectancy. This leads Ghislandi et al. [21] to note that ‘if a poor country decided to use the HDI to determine its stage of development, income would count much more than life length’ (p.3). Indeed, their comparison of trade-offs between countries question the extent to which such trade-off values are meaningful at all in terms of modes of ‘increasing development’ in a holistic manner in any country of the world.

A final criticism lies in the fact that GNI, education and life expectancy are empirically and conceptually strongly correlated with each other [24] through what we might term the ‘socioeconomic gradient’ [25]. In this sense, then, the composite index that is the HDI may not in fact be revealing much more than its component parts.

Reflecting on this, by not addressing some of these criticisms, the generated HDIs at the sub-national level—or, indeed, other measurements based upon the core HDI components such as the Inequality-adjusted HDI—simply reflect the limitations of the core national measures. Indeed, it could be argued that without explicitly addressing some of the challenges of measurement error, data quality and consistency across time and space, sub-national HDI measures may in fact only serve to exaggerate these problems.

### The Human Life Indicator

In 2019, Ghislandi et al. [21] proposed a new ‘measure of human development’, namely the ‘Human Life Indicator’. Based upon the principle of attempting to ‘keep it simple’ in order to reduce the redundancies of a multidimensional index and to ease access to underlying consistent data, this measure is grounded in just one measure—namely life expectancy. As discussed above, Ghislandi et al. [21] argue that the strong relationship between the components of the HDI renders the information loss entailed by focusing on just one component minimal. They also observe that a focus on one area (namely life expectancy) addresses the critiques of HDI above by averting the need to depend on some (arbitrary) trade-offs; reporting lower measurement errors than, say, measures of GNI per capita (as demonstrated by Wolff, Chong, and Auffhammer [20]; and is built upon data which is more comparable and consistent across time and space. Finally, we observe that Sen [39] explicitly suggests that mortality should be considered as an indicator of economic success and failure.

The HLI is, however, an adaptation of the standard measure of life expectancy. As Ghislandi et al. [21] observe, life expectancy at birth is the arithmetic average of ages of death and, as such, is not influenced by the distribution of ages of death around that average. As a consequence, two countries with the same life expectancy at birth may have very different infant mortality figures. At the most basic level, if people experience the age-specific survival rates in a life table, they will die at various ages. The arithmetic mean of their lifespans is their life expectancy at birth. The HLI, then, is the geometric average of those lifetimes. The value in using the geometric mean is that is, effectively penalizes countries which have a relatively high variation in the length of lives. The justification for this is, again, grounded in the strong association between infant and child mortality rates and education. An explicit assumption is that inequality in mortality is broadly a reflection of inequalities in society; and that reducing such inequalities is a core development goal. In essence, an economy with many disadvantaged people dying young and many richer people dying old is considered to be ‘less developed’ than an economy where most of the population live to the about the same age. As such, the HLI treats the reduction in the inequality in lifetimes as an additional contributor to the improvement in human development. The endpoint of the HLI is, effectively, where HLI = life expectancy at birth; i.e. where there is little or no infant, child or middle-age mortality.

A robustness check was performed to explore the correlation between the new HLI and the HDI (and its component parts). Even though the HLI is much simpler, the correlation to the 2016 HDI data was 0.93. It can, therefore, be argued that the HLI does, indeed, represent a ‘new, simple measure of human development’ which is largely free of the concerns of the HDI relating to data and consistency. The publication of the new world rankings of HLI in 2018 attracted a significant degree of media interest [26]. In particular, the observation that Hong Kong, rather than Norway, topped the rankings garnered much attention [27].

### Sub-national Human Life Indicators

In the discussion above, we identified the value of determining sub-national indicators of development in order to gauge the degree of inequality within countries and, hence, the challenges ahead in terms of resolving these differences *within* countries as part of the SDG agenda. Although sub-national HDI measures exist, without addressing the core challenges of using the HDI, especially in terms of measurement error and consistency over time and space, these new measures are still susceptible to the same critique. As Ghislandi et al. [21] observe, ‘HLIs can be calculated whenever life tables are available, including places where suitable data on education and economic output per capita are not available’. This means that for countries where sub-national life tables exist, it is therefore possible to generate HLIs. Indeed, using the Indian Sample Registration System, Ghislandi et al. [21] were able to generate HLIs for the states of India for two times points between 2007 and 2015 (as well as urban and rural differentials). Their observations of the changing pace of development in states (and differentiated by rural and urban areas) could, they argued, be ‘potentially useful in assessing the sustainable development goal of reducing regional inequalities’ (p.11).

At the national level, long runs of life tables from the past are available, either from the UN [28], WHO [29], national or supranational statistical agencies (such as Eurostat) and, of course, the Human Mortality Database [30]. Indeed, extant data from the Human Mortality Database allowed Ghislandi et al. [21] to generate a consistent time series of HLI for Italy, Finland, the Netherlands and Sweden from the late-nineteenth century through to the present day. At the sub-national level, however, such long-runs of life tables are much rarer. Most publicly available sub-national life tables only cover a short time span in the recent past (such as the case for India as reported in Ghislandi et al. [21], and also, for example, in New Zealand [31]). While these undoubtedly give an important snapshot of regional inequalities in development in the recent past, they are not necessarily able to represent change over time. We address these challenges in the conclusion. In this paper, we attempt to provide ‘proof of concept’ by generating HLIs for the states of the United States of America. The reason for choosing this setting is that it has the longest available run of sub-national life tables upon which to perform the analysis and present data for both contemporary states as well as change over time. In other words, it allows us to see the most that could be possible if we apply HLI as a complimentary development indicator from today onwards—especially in the context of ever more countries developing Civil Registration and Vital Statistics [CRVS] systems [32]. In doing so, we hope to answer the following questions: empirically, what does the HLI tell us about sub-national differences in development (and, by definition, mortality and inequality) in the United States. By exploring the relative differences in HLI between states over time, we can then determine the extent to which inequality (of development) is increasing or decreasing over time. Methodologically, following Ghislandi et al. [21] we will then explore the correlation between the HLI and the HDI. The reason for doing this is that given the familiarity of the HDI as a cornerstone of measuring development, and one which broadly accords with popular *expectations*, if we can show that a simpler measure (and one with fewer potential internal challenges) broadly accords to it, then it can strengthen the argument for the HLI to be a worthy addition to the battery of measures used to measure development.

## Materials and methods

In this paper, we endeavor to deliver a ‘proof of concept’ by systematically calculating HLIs for the States of the United States of America for the period 1959 to 2016. Firstly, life tables were downloaded from the United States Mortality Database [33]. The calculation of the HLI is presented in the Introduction in qualitative form. Formally, however, it is calculated as follows:

$$HLI=\prod_{i=1}^{N}({age}_{i}+a^{i})d_{i}$$

where *age_i_* is the age at the lower end of the age interval *i* in a life table, *a^i^* is the average number of years lived in the interval by those who die in the interval, *d_i_* is the fraction of deaths in age interval *i* among all deaths, and *N* is the number of age intervals in the life table. The calculations were performed in R.

To answer our empirical research questions, we will first produce HLIs for the 50 states of the USA for the period 1959 to 2016. In the introduction, we stated that the logical endpoint of HLI is that it should be equal to life expectancy at birth. As such, we then simply perform the following calculation:

$$Inequality\;of\;distribution\;of\;mortality\;across\;life\;course=\frac{\left(e_{0}-HLI\right)}{e_{o}}$$

where *e_o_* is life expectancy at birth, in order to ascertain the relative contribution of inequality in ages of death (which are effectively penalizing the given state’s HLIs most). In order to gauge the extent to which these degrees of inequality have increased or declined over time—as a proxy for determining progress towards SDG-10 on reducing inequalities *within* countries, we perform a simple analysis of convergence of HLI over time. We address our core methodological question of the possibility of HLI being a reasonable substitute for HDI by performing a simple correlation analysis of the most recent HLI calculated by us and the respective sub-national HDIs for US States as calculated by Smits and Permanyer [15].

## Results and discussion

**Fig 2:**
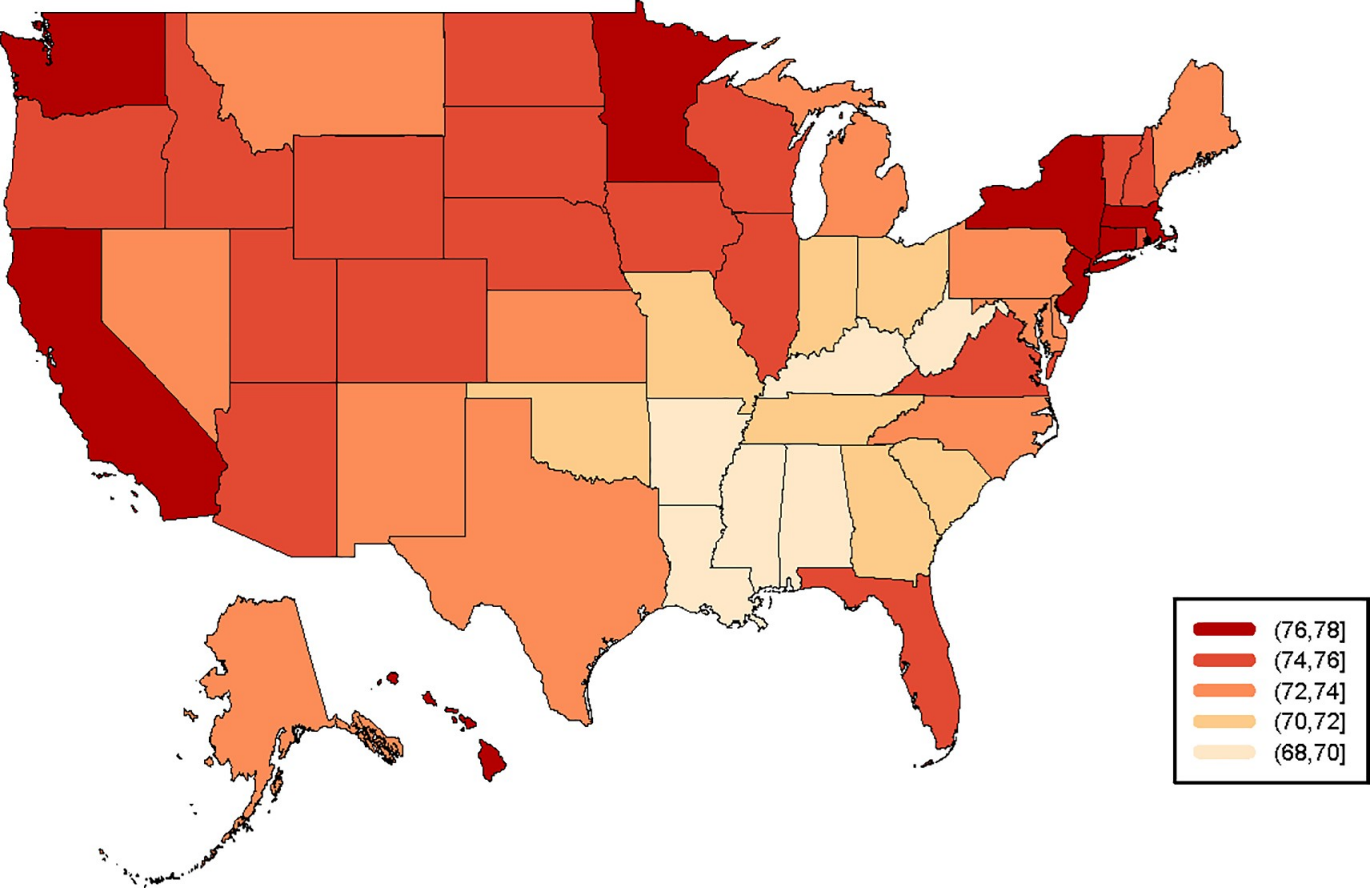
HLI of states of the United States of America, 2016. Source: Authors’ calculations based on data derived from United States Mortality Database [33].

**Fig 3:**
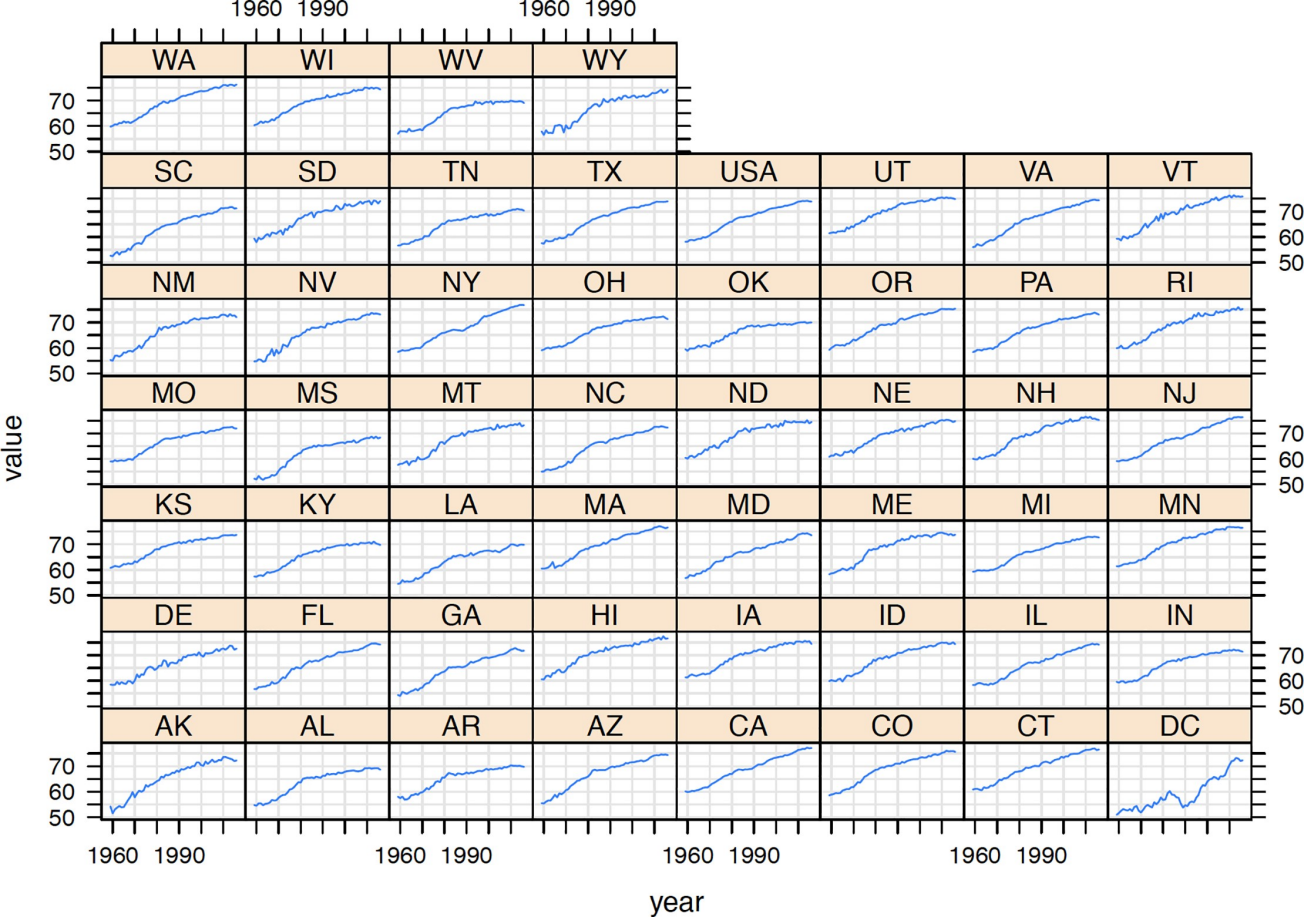
HLI of states of the United States of America, 1959–2016. Source: Authors’ calculations based on data derived from United States Mortality Database [33].

Fig 2 represents the most recent sub-national HLIs to be calculated for the states of the United States (namely for 2016). The range clearly justifies the exercise in terms of generating sub-national HLIs. While the national HLI in 2016 is 73.9, the range is 68.4 (Mississippi) through to 77.2 (California). The five states with the highest and lowest HLIs respectively are presented in Table 1. The complete ranking for 2016 is presented in Appendix I. Together the data show significant heterogeneity, but also reveal certain geographical clusters. The lowest HLIs are to be found in the Southeastern states (except for Florida and Virginia). Lower HLIs are also to be found in Oklahoma, Missouri, Indiana and Ohio. On the contrary, the highest HLIs are to be found in in the north-east, Minnesota and the Pacific Coast. Unsurprisingly, these HLIs broadly correlate with rankings for various measures of income (including earnings, income inequality & poverty) [34]; health [35]; and educational attainment [36]. Fig 3 shows the changes in HLI over time for all states during the period 1959–2016. With this overview, we also see that a general trend of improvement masks significant differences in the past and uneven patterns of development. Consider Alaska and Arizona, for example, who transitioned from one of the lowest HLIs in 1960 to among the highest by 2016. Other states who had roughly the same HLIs in 1960, meanwhile, show diverse patterns today—compare Washington to Oklahoma, for example, which has seen improvements stagnate since 2016. Washington, DC can be identified as an outlier, with quite a unique history compared to the other states—a reflection of the challenging health, poverty and inequality environment of the area [37,38].

**Table 1:** Ranked bottom and top five states, HLI, 2016.

*Lowest*		*Highest*	
State	HLI 2016	State	HLI 2016
Arkansas	69.8	California	77.2
Kentucky	69.7	New York	76.8
West Virginia	69.0	Hawaii	76.6
Alabama	68.6	Massachusetts	76.6
Mississippi	68.4	Connecticut	76.6

Source: Authors’ calculations based on data derived from United States Mortality Database [33]

**Fig 4:**
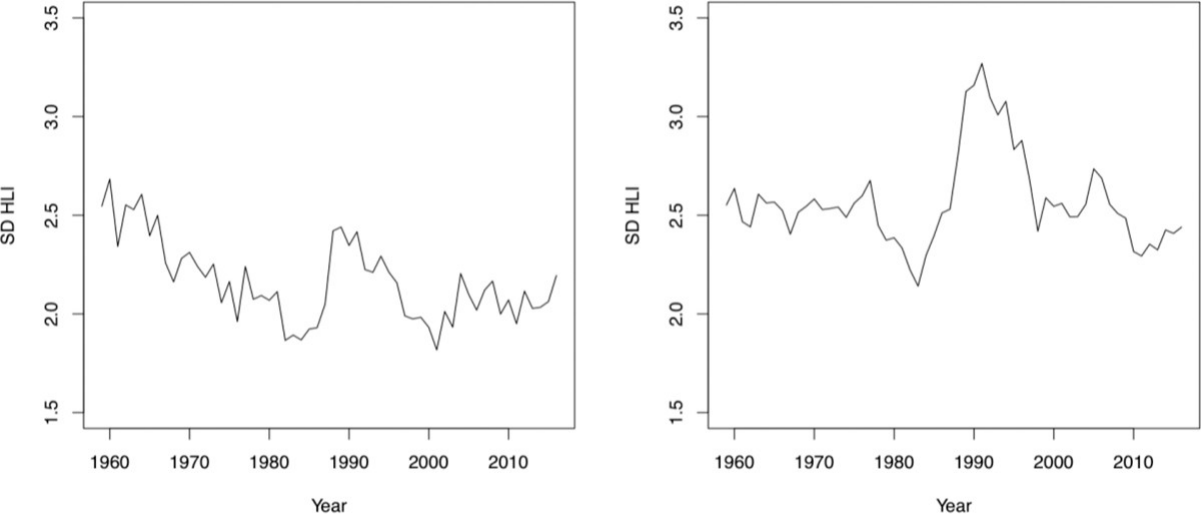
Standard deviation of HLIs for US states, **(a)** female [left], **(b)** male [right]. Source: Authors’ calculations based on data derived from United States Mortality Database [33].

Fig 4(A) and 4(B) represents the standard deviation of the distribution of HLIs by states over the period 1959–2016 for females and males respectively. This is a means of exploring changing patterns of the heterogeneity of development across time. Because this is a measure which, after Sen [39], places mortality at the heart of the determination of development, it is acutely sensitive to mortality crises. This is clearly evident in the period from the mid-1980s through to the late-1990s. This can be associated with the changing incidence of AIDS; with the peak of new diagnoses was associated with the expansion of the AIDS surveillance case definition in the early 1990s, followed by sharp declines in AIDS incidence and deaths from around 1996 [40]. Deaths attributable to AIDS were not evenly distributed according to the population across the United States either over this period [40] or, for that matter, either death from AIDS or HIV diagnoses today [41]. Without the spike associated with AIDS and a decline of variation between 1960–1980 among females, it is difficult to discern any particular pattern of convergence among states concerning the HLI. This is, again, likely due to the ongoing variation in various background measures of income, education and other indicators on the ‘socioeconomic gradient’ [25] as well as an unequal distribution of mortality; whether this is lingering inequalities in measures such as infant mortality [42,43] or newer developing modalities of death such as the opioid crisis [44,45] - all with familiar socioeconomic determinants.

**Fig 5:**
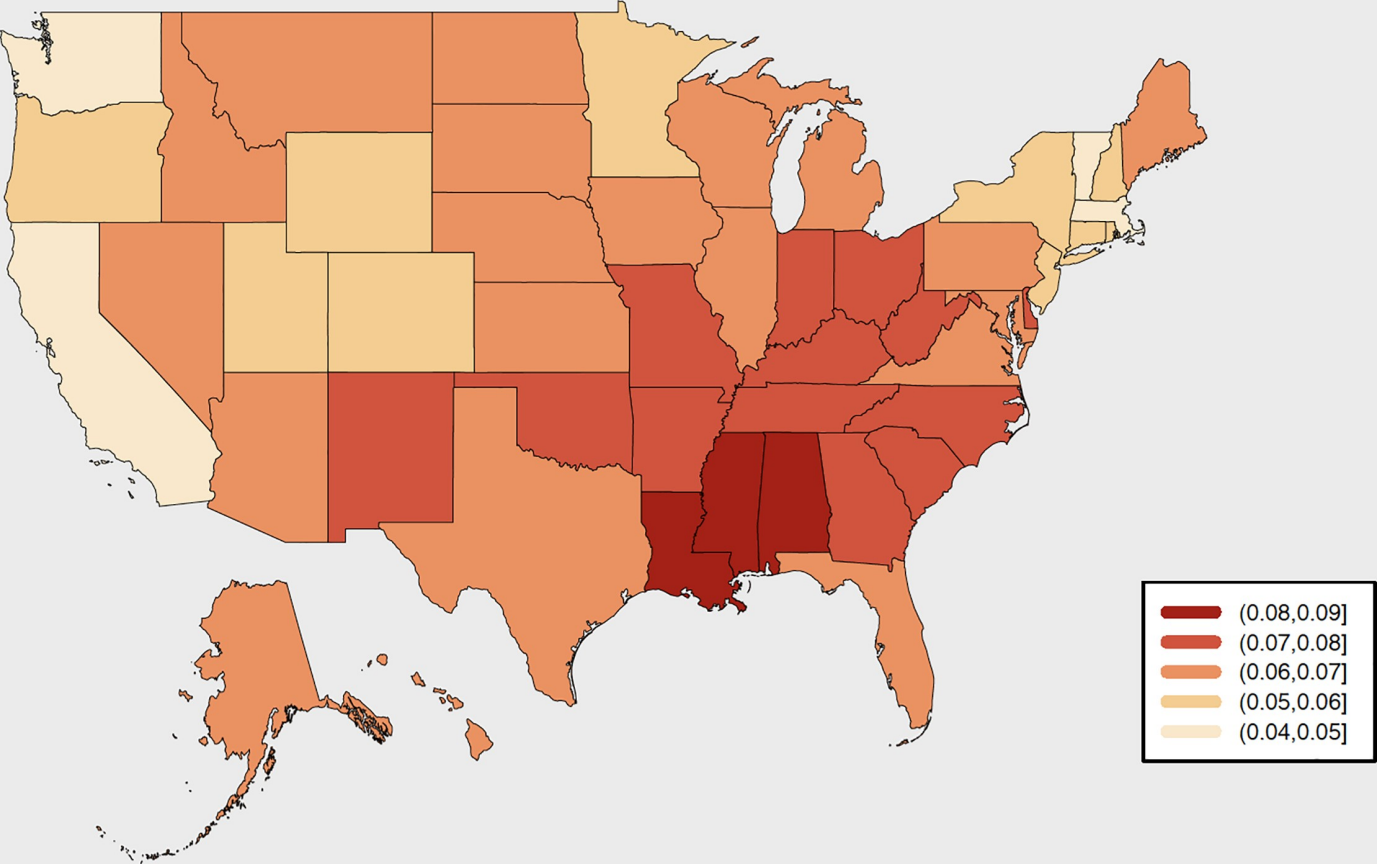
Inequality of experience of death across the life-course HLIs for US states, 2016. Source: Authors’ calculations based on data derived from United States Mortality Database [33].

As noted in the Introduction, if we are interested in inequalities in the distribution of mortality across the life-course, it is important to supplement the standard HLI with an understanding of the relative importance of these inequalities. Fig 5 represents life expectancy at birth (minus HLI) divided by life expectancy at birth for the states of the USA in 2016. As a reminder, if these figures were zero then all deaths would be occurring at the age of life expectancy at birth. The higher the figure, the greater the distribution of death across the life course and, hence, the more unequal the experience of mortality is. As Fig 5 demonstrates, inequality of the mortality experience across the life-course is greatest in Louisiana, Mississippi and Alabama. The lowest, at roughly half that of the states just mentioned, can be found in California, Washington, Massachusetts and Vermont. Again, this broadly maps onto the ‘socioeconomic gradient’ of health inequalities referred to earlier [25].

**Fig 6:**
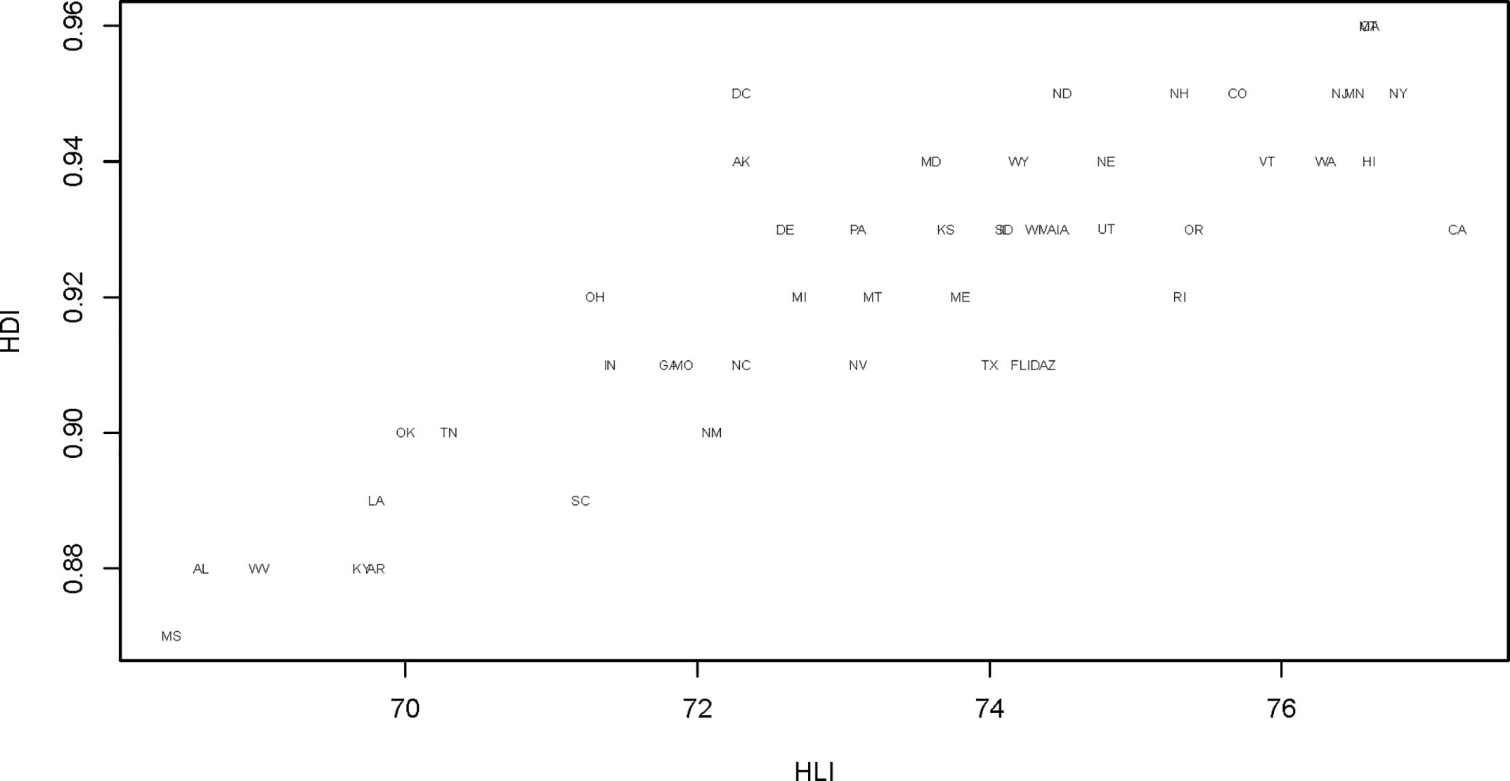
Correlation of HLI and HDI for US states, 2016. Source: Authors’ calculations based on data derived from United States Mortality Database [33].

Finally, we explored the correlation between the HLI and the sub-national HDI for the latest date for which both measures are available, namely 2016. We found that the correlation coefficient between HDI and HLI for 2016 was 0.87. This correlation is represented graphically for all states in Fig 6.

## Conclusions

The HDI is regarded as the ‘go-to’ measure for benchmarking progress in human development. However, the Index has been subject to a wide number of criticisms relating to consistency over time, measurement error and questionable trade-offs between elements. Furthermore, it has been argued that interconnectedness of the indicators used to make up the index may mean that the difficulties of obtaining reliable multidimensional datasets to compute the index are surplus to actual requirement. While the generation of sub-national HDIs allows for a more nuanced view of development within countries—and allow us to better approach SDG-10 to reduce inequalities between and *within* countries—by not addressing these key criticisms of the HDI the value of these figures is still limited.

Against this context, then, Ghislandi et al. [21] proposed the HLI—a simple measure based upon the assumption of inequality of mortality being a core measure of development [39] which, in turn, can be qualitatively linked through to the other indicators which make up the HDI. The working through of the US case study in this paper cements the idea that the HLI represents a simple, transparent measure of development (which includes a consideration of inequality). Empirically, the data show that for the United States, there is a significant degree of heterogeneity *within* the country in terms of the distribution of mortality across the life course, with the difference between the states with the highest and lowest inequalities of mortality distribution across the life course being a factor of three.

Methodologically, the data we have presented correlates strongly to the published HDIs generated by Smits and Permanyer [15] and also passes our ‘expectation’ test in terms of which states score highly and which score less well. These figures are calculated on a much simpler roster of data; are easier to interpret and explain; and are not subject to the main critiques of HDI relating to trade-offs, consistency of calculation over time, and (to a much greater extent) measurement error.

There are numerous limitations with the HLI approach. In common with the HDI, for example, issues relating to disability and ‘healthy life expectancy’ are not considered. Of course, the greatest limitation of the generation of sub-national HLIs relates to the available of sub-national life tables. Currently, there is little doubt that only a small number of countries in the world—and only a handful outside of the Global North—have even modest runs of such life tables to be able to generate such data. Of course this, in turn, is grounded in the global underdevelopment of Civil Registration and Vital Statistics [CRVS] systems around the world [46]. Of course, the quality of the regional vital statistics and population counts/estimates as well as the size of the regional administrative/geographical populations themselves affect the reliability of the existing sub-national life tables and therefore influence the reliability of the estimated HLIs. However, in recent years the recognition of the need to develop and improve such systems for a variety of reasons, including monitoring the SDGs has been strongly recognized [47,48]. In response, more and more countries are actively developing policies to develop their CRVS systems [32]. Similarly, with UN support, census systems are being expanded throughout the world [49,50,51]. If these developments continue, therefore, we can expect that ever more countries will have the data capacity to generate sub-national life tables upon which HLIs can be calculated. The data requirements of the HDI are much more intensive than those of the HLI; furthermore, it has been demonstrated by Ghislandi et al. [21] that the measurement error of life expectancy is significantly lower than that of the other measures of education and GNI per capita required to compute the HDI. We may assert, then, that as CRVS systems expand around the world, compared to the HDI the HLI represents a simpler, more transparent, more consistent, and more reliable means of measuring development and inequality both across and within countries.

## Supporting information

S1 Appendix Full rankings of US States by HLI, 2016 [ranked from lowest to highest]. (DOCX)
