# Supplementary material for: Measuring inequalities of development at the sub-national level: From the human development index to the human life indicator
Source: PLoS One. 2020 Apr 30;15(4):e0232014. doi: 10.1371/journal.pone.0232014 (PMC7192420; doi:10.1371/journal.pone.0232014)
Supplement: S1 Appendix — (DOCX) [file pone.0232014.s001.docx]

**Appendix a: Full rankings of US States by HLI, 2016 [ranked from lowest to highest]**

| State | HLI 2016 | State | HLI 2016 |
| --- | --- | --- | --- |
| California | 77.2 | Maine | 73.8 |
| New York | 76.8 | Kansas | 73.7 |
| Hawaii | 76.6 | Maryland | 73.6 |
| Massachusetts | 76.6 | Montana | 73.2 |
| Connecticut | 76.6 | Nevada | 73.1 |
| Minnesota | 76.5 | Pennsylvania | 73.1 |
| New Jersey | 76.4 | Michigan | 72.7 |
| Washington | 76.3 | Delaware | 72.6 |
| Vermont | 75.9 | District of Columbia | 72.3 |
| Colorado | 75.7 | North Carolina | 72.3 |
| Oregon | 75.4 | Alaska | 72.3 |
| Rhode Island | 75.3 | New Mexico | 72.1 |
| New Hampshire | 75.3 | Missouri | 71.9 |
| Utah | 74.8 | Georgia | 71.8 |
| Nebraska | 74.8 | Indiana | 71.4 |
| North Dakota | 74.5 | Ohio | 71.3 |
| Iowa | 74.5 | South Carolina | 71.2 |
| Virginia | 74.4 | Tennessee | 70.3 |
| Arizona | 74.4 | Oklahoma | 70.0 |
| Idaho | 74.3 | Louisiana | 69.8 |
| Wisconsin | 74.3 | Arkansas | 69.8 |
| Wyoming | 74.2 | Kentucky | 69.7 |
| Florida | 74.2 | West Virginia | 69.0 |
| Illinois | 74.1 | Alabama | 68.6 |
| South Dakota | 74.1 | Mississippi | 68.4 |
| Texas | 74.0 |  |  |
